# Supplementary material for: Morphological and Molecular Diversity among Pin Nematodes of the Genus Paratylenchus (Nematoda: Paratylenchidae) from Florida and Other Localities and Molecular Phylogeny of the Genus
Source: Plants (Basel). 2023 Jul 26;12(15):2770. doi: 10.3390/plants12152770 (PMC10421193; doi:10.3390/plants12152770)
Supplement: Supplementary file 1 [file plants-12-02770-s001.zip › plants-2426459-supplementary.pdf]

Article

Morphological and molecular diversity among pin nematodes of the genus *Paratylenchus* (Nematoda: Paratylenchidae) from Florida and other localities, and molecular phylogeny of the genus

Sergio Álvarez-Ortega [1,*], Sergei A. Subbotin [2,3], Koon-Hui Wang [4], Jason D. Stanley [5], Silvia Vau [5], William Crow [6] and Renato N. Inserra [5]

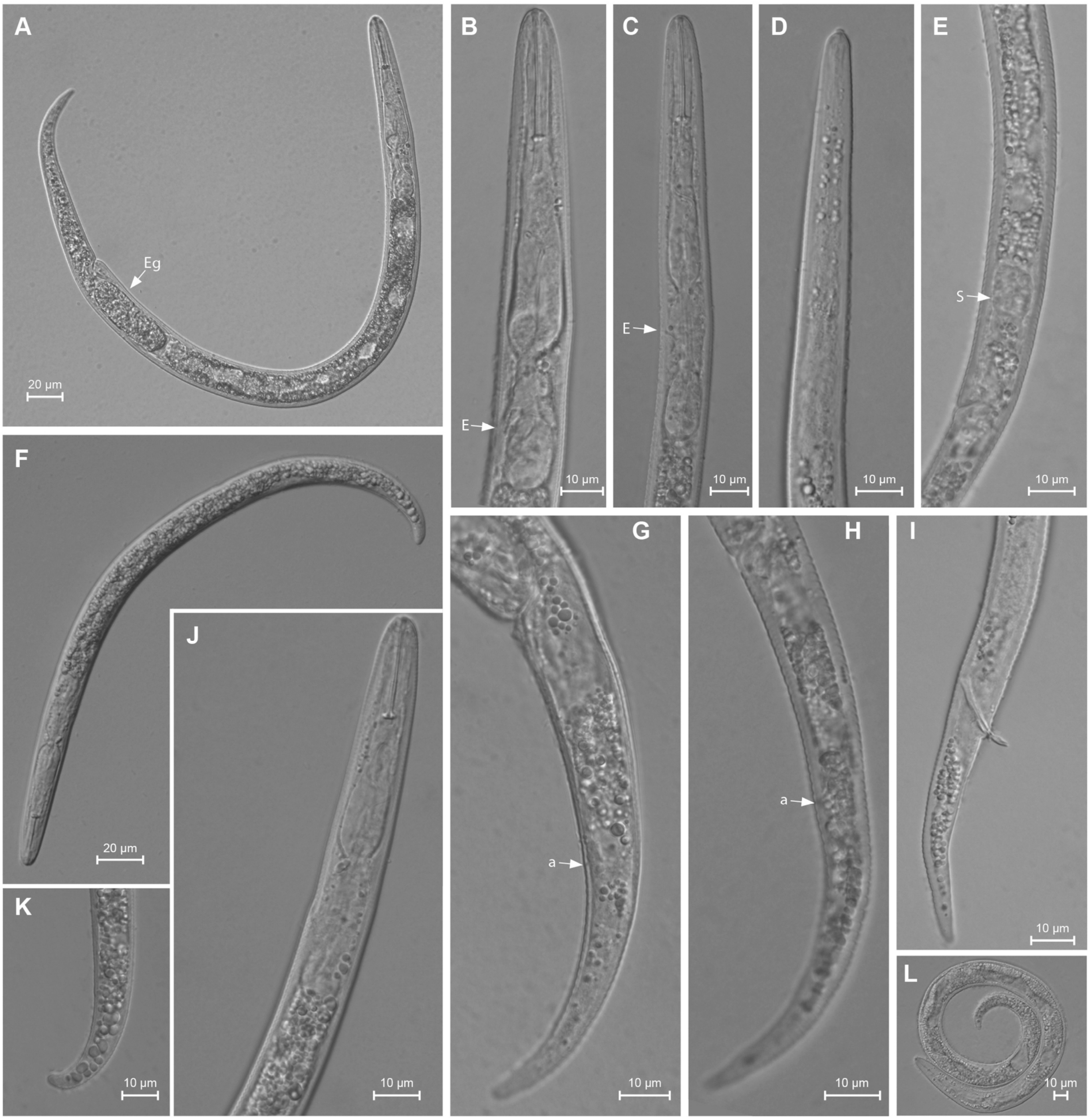

Figure S1. Light microscopic photos of *Paratylenchus* from *Zoysia* sp. in Florida (CD3651). A: Entire body of an egg-laying female. Note egg (arrowed); B, C: Anterior bodies of an egg-laying and immature female, respectively. Note excretory pore (arrowed); D: Anterior body of male; E: Vulvar region showing a prominent spermatheca (arrowed); F Second stage juvenile entire body; G, H: Posterior bodies of egg-laying and immature female. Note anus (arrowed); I: posterior body of male; J: Anterior and posterior body of J2; K: Coiled fourth stage juvenile entire body.

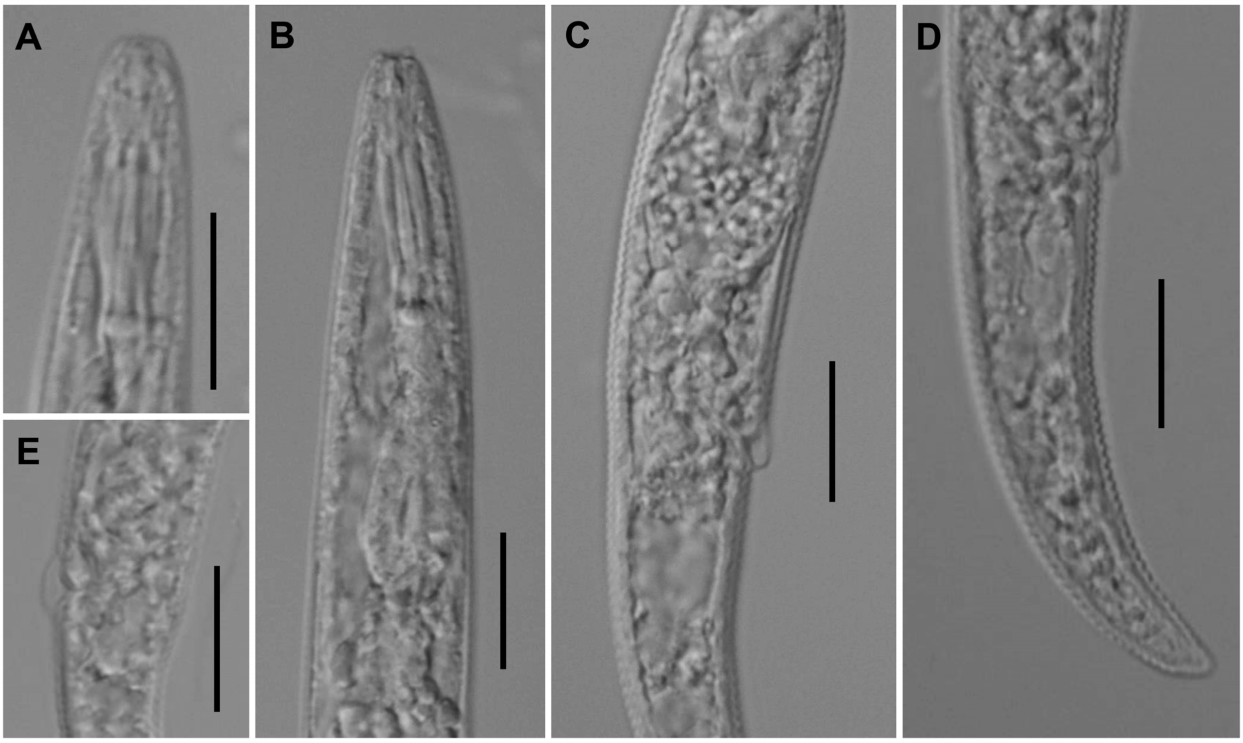

Figure S2. Light microscopic photos of *Paratylenchus minutus* (*P. shenzhenensis* syn. n.) female from *Coffea* sp. in Hawaii. A: Truncate lip region and stylet; B: Pharyngeal region; C: Posterior genital tract with large spermatheca; D: Posterior body; E: Vulvar region. (Scale bars = 10 μm).

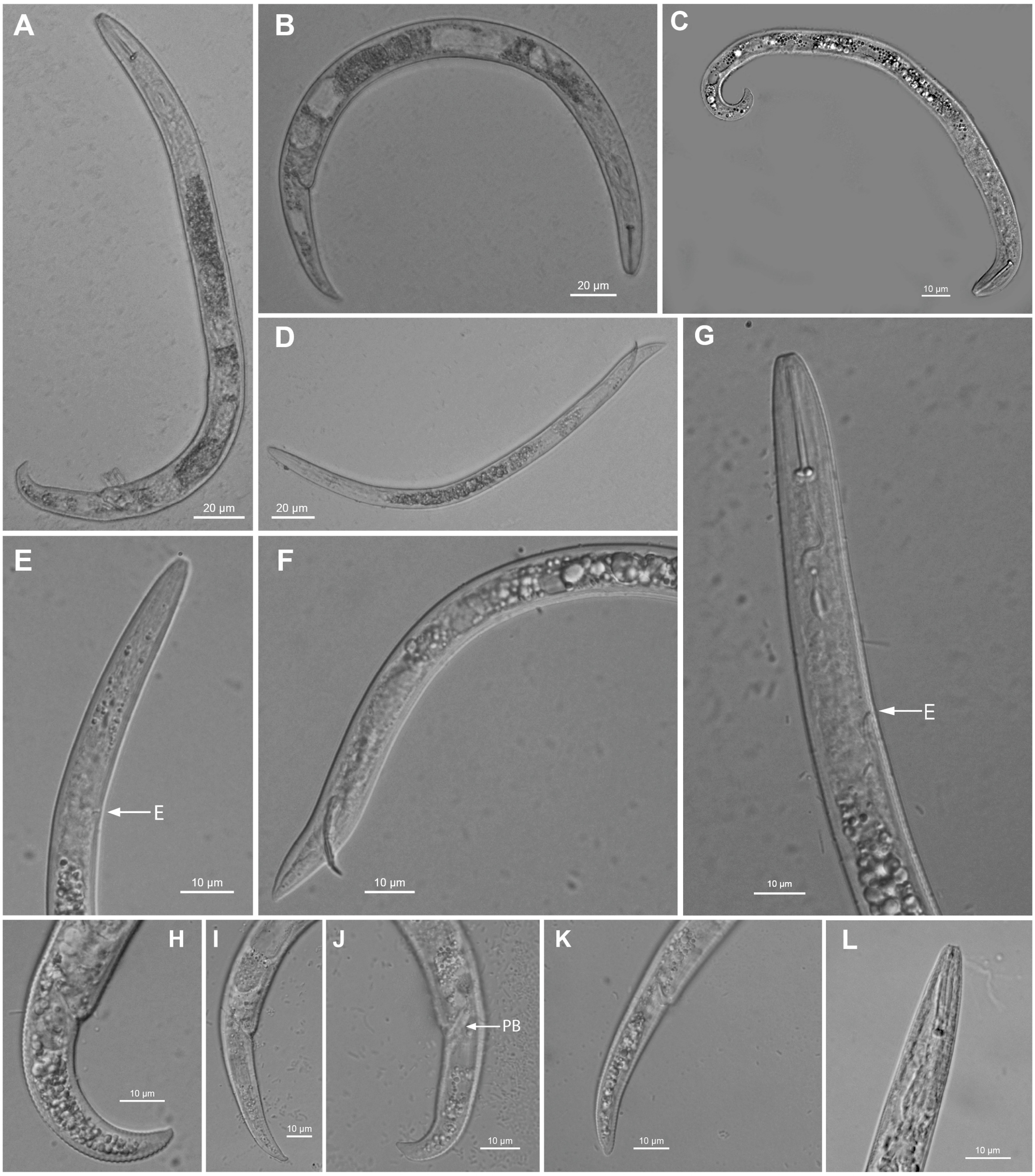

Figure S3. Light microscopic photos of *Paratylenchus minutus* (*P. shenzhenensis* syn. n.) female, male and fourth stage juvenile from *Hemerocallis* sp. in Florida. A, B: Entire body of female; C: Entire body of fourth stage juvenile; D: Entire body of male; E, F: Anterior and posterior body of male. Note excretory pore (arrowed); G: Pharyngeal region of female showing a truncate lip region and excretory pore (arrowed); H-K: Shape variations of female posterior body. Note large spermatheca and rudimentary post-vulvar branch (PB); L: Anterior body with truncate lip region of a female *P. minutus* from *Coffea* sp. in Hawaii is included for comparison.

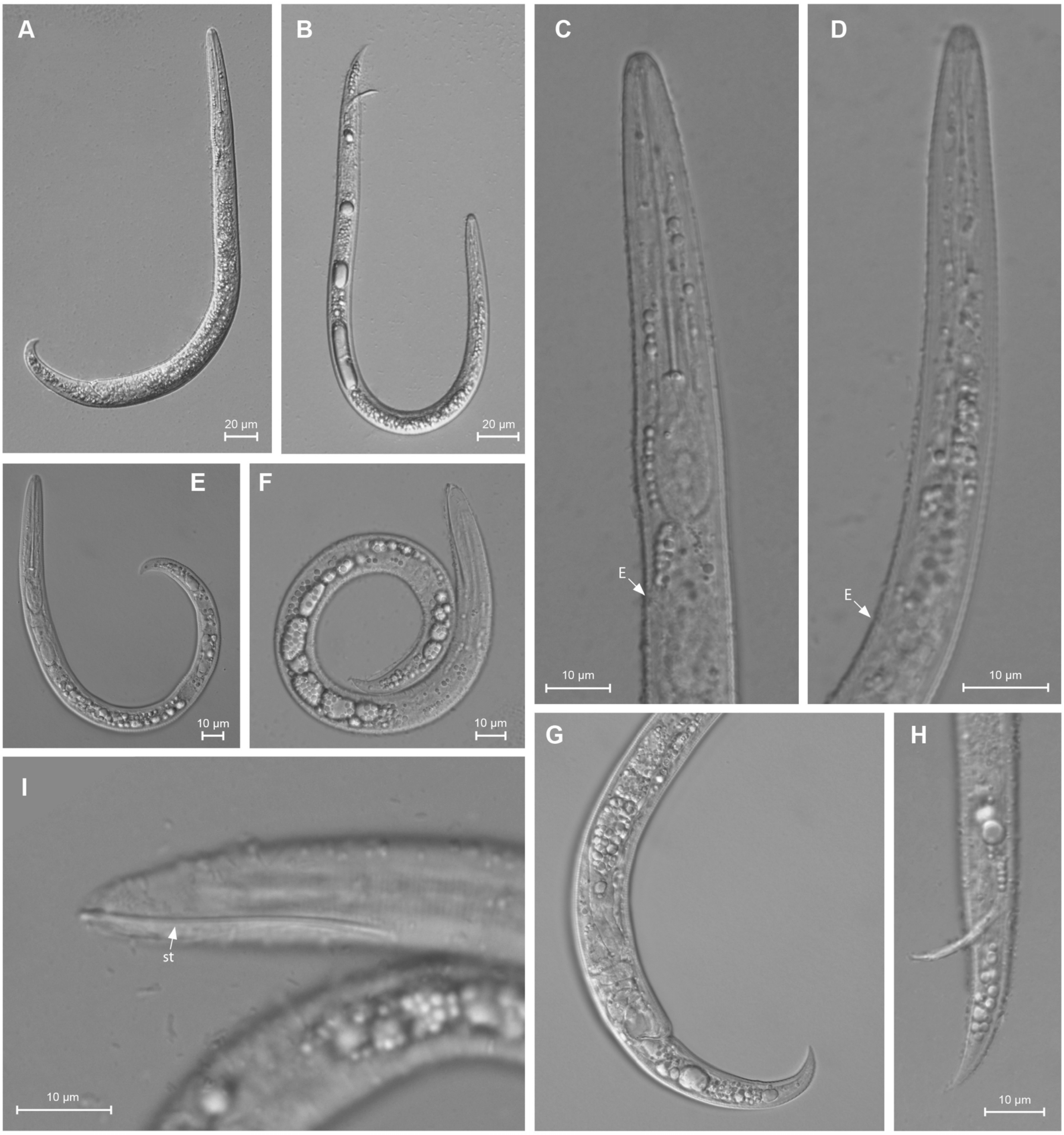

Figure S4. Light microscopic photos of *Paratylenchus straeleni* from *Quercus virginiana* in Florida. A, B: Female and male entire bodies, respectively; C, D: Female and male anterior bodies, respectively. Note excretory pore (arrowed); E: Second stage juvenile entire body; F: Fourth stage encased in the molted cuticles of J2 and J3; G: Posterior body of female showing oocytes and crustaformeria; H: Posterior body of male; I: Magnified figure F showing the molted stylet (arrowed) of J2 adhering to the molted cuticle.

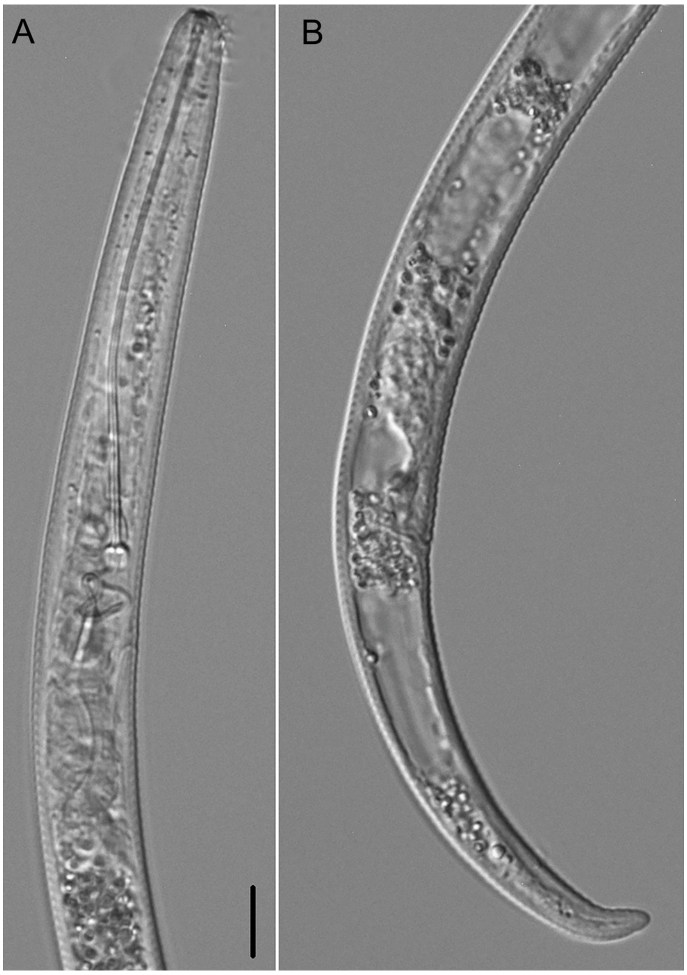

Figure S5. Light microscopic photos of *Paratylenchus* sp. FL2 female from palm in Florida. A: Anterior body region; B: Posterior body region (Scale bars = 10 μm).

Table S1. Species of the pin nematodes reported in Florida.

Species	Morphological or morphometrical characterization	Molecular characterization	Identification without any published morphological datasets	Reference
P. aciculus	-	-	+	Raski (1976); Esser (1992); Lehman (2002)
P. acti	+	+	-	This study
P. aculentus	-	-	+	Esser (1992); Lehman (2002)
P. alleni	-	-	+	Esser (1992); Lehman (2002)
P. aquaticus	+	+	-	This study
	-	-	+	Esser (1992); Lehman (2002)
P. audriellus	-	-	+	Esser (1992)
P. besoekianus	-	-	+	Lehman (2002)
P. breviculus	-	-	+	Esser (1992); Lehman (2002)
P. colbrani	-	-	+	Esser (1992); Lehman (2002)
P. costatus	-	-	+	Lehman (2002)
P. curvitatus	-	-	+	Esser (1992); Lehman (2002)
P. dianthus	-	-	+	Esser (1992); Lehman (2002)
P. elachistus	+	-	-	Steiner (1949); Raski (1975a)
P. elegans	+	-	-	Raski (1962)
P. enatus	-	-	+	Lehman (2002)
P. goldeni	+	+	-	This study
	-	-	+	Esser (1992); Lehman (2002)
P. goodeyi	-	-	+	Esser (1992); Lehman (2002)
P. hamatus	-	-	+	Esser (1992); Lehman (2002)
P. hawaiiensis sp. n.	+	+	-	This study
	-	+	+	Singh *et al*. (2021)
P. holdemani	-	-	+	Lehman (2002)
P. leiodermis	-	-	+	Esser (1992); Lehman (2002)
P. leptos	-	-	+	Esser (1992); Lehman (2002)
P. marylandicus	-	-	+	Esser (1992); Lehman (2002)
P. microdorus	-	-	+	Esser (1992); Lehman (2002)
P. mimulus	-	-	+	Esser (1992); Lehman (2002)
P. minor	-	-	+	Lehman (2002)
P. minusculus	+	-	-	Tarjan (1960)
P. minutus	+	+	-	This study
	-	-	+	Esser (1992); Lehman (2002)
	-	+	+	Singh *et al*. (2021)
P. nainianus	-	-	+	Raski (1975b)
P. nanus	-	-	+	Lehman (2002)
P. nawadus	-	-	+	Lehman (2002)
P. oostenbrinki	-	-	+	Lehman (2002)
P. paralatescens (in Troccoli *et al*. (2002) as *P. latescens*)	+	+	-	Troccoli *et al*. (2002)
	+	+	-	This study
P. peperpotti	-	-	+	Esser (1992); Lehman (2002)
P. peraticus	-	-	+	Esser (1992); Lehman (2002)
P. perlatus	-	-	+	Lehman (2002)
P. pesticus	-	-	+	Esser (1992); Lehman (2002)
P. projectus (= *P. amblycephalus*)	-	-	+	Esser (1992); Lehman (2002)
P. roboris sp. n.	+	+	-	This study

P. robustus	-	-	+	Lehman (2002)
P. salubris	-	-	+	Lehman (2002)
P. sarissus	+	-	-	Tarjan (1960)
	-	-	+	Esser (1992)
P. sheri	-	-	+	Lehman (2002)
P. straeleni	+	+	-	This study
	-	-	+	Lehman (2002)
P. vandenbrandei	-	-	+	Esser (1992); Lehman (2002)
P. yokooi	-	-	+	Lehman (2002)

Table S2. Morphometrics of *Paratylenchus aquaticus* from the original description in Ivory Coast, compared to those of a population of *P. aquaticus sensu lato* from Mexico (Brzeski, 1995) and another from Kansas (classified as type B in Van den Berg *et al.*, 2014).

**Population**	Paratypes Ivory Coast		*P. aquaticus* Mexico		*P. aquaticus* type B Kansas
**Character**	♀ (fixed)	♂ (fixed)	♀ (fixed)	♂ (fixed)	♀ (fixed)
n	22	4	31	?	7
L	340 (270-370)	300 (290-310)	331.0 ± 213.4 (303-390)	-	301.0 ± 36.5 (272-378)
Stylet length (St)	16.0-20.0	-	15.8 ± 0.6 (14.5-17.5)	-	17.0 ± 1.0 (16.0-18.5)
Conus length	-	-	-	-	10.0 ± 0.7 (9.0-11.0)
Stylet shaft + knob height	-	-	-	-	7.0 ± 0.8 (6.0-8)
Knob width	-	-	-	-	3.0 ± 0.5 (3.0-3.5)
Knob height	-	-	-	-	2.0 ± 0.3 (1.5-2.0)
DGO	4.0-8.0	-	-	-	3.5 ± 0.3 (3.0-3.5)
Median bulb valve length	-	-	-	-	-
Median bulb width	-	-	-	-	-
Isthmus length	-	-	-	-	-
Pharynx length	85.4	62.0	-	-	71.0 ± 4.4 (66.0-78.0)
Anterior end to excretory pore (Ep)	-	-	71.0 ± 4.2 (64.0-81.0)	-	62.5 ± 4.7 (58.0-72.0)
Max body width	12.1	11.1	-	-	14.5 ± 2.2 (11.0-17.0)
Body width at vulva	-	-	-	-	-
Body width at anus	-	-	-	-	-
Anterior end to median bulb base	-	-	-	-	-
Lateral field width	-	-	-	-	2.5 ± 0.2 (2.0-2.5)
Anterior end-vulva distance	-	-	-	-	-
Vulva-tail terminus distance	-	-	-	-	-
Genital tract length	-	-	-	-	-
Vulva-anus distance	-	-	-	-	30.5 ± 11.3 (29.5-35.5)
Tail length	20.0-24.0	21.0-23.0	-	-	23.5 ± 1.9 (22.0-26.5)
Stylet base (Stb) to median bulb valve base (v)	-	-	-	-	-
Spicule length	-	21.0-22.0	-	16.0-18.0	-
Gubernaculum length	-	-	-	3.0-4.0	-
PERCENTAGES					
V	79.0-84.0	-	81.3 ± 0.9 (79.0-84.0)	-	80.5 ± 1.1 (79.5-82.5)
G or T	23.0-31.0	-	-	-	-
Stb-v/St	-	-	-	-	-
St/L	-	-	-	-	-
Ep/L	-	-	21.5 ± 0.8 (18.5-23.5)	-	20.9 ± 1.2 (19.1-22.2)
RATIOS					
a	30.0 (26.0-39.0)	28.0 (26.0-30.0)	32.1 ± 2.0 (27-35)	-	20.9 ± 2.5 (18.5-35.7)
b	4.1 (3.5-4.7)	4.9 (4.8-5.0)	3.9 ± 0.3 (3.5-4.8)	-	4.2 ± 0.5 (3.6-4.8)
c	15.0 (14.0-18.0)	14.0 (13.0-15.0)	15.3 ±1.3 (13.2-18.0)	-	12.8 ± 0.9 (11.3-14.3)
c′	-	-	3.1 ± 0.3 (2.6-3.7)	-	2.8 ± 0.3 (2.4-3.1)

Table S3. Morphometrics of males and J2 of *Paratylenchus goldeni* associated with *Zoysia* sp. in Florida.

**Population**	Madison, Madison County (CD3651 – N21-01426-4)		
**Character**	♂ (live)	♂ (fixed)	J2 (live)
n	5	1	3
L	405 ± 34.1 (341.5-439.5)	356.5	316.1 ±16.9 (279.9-338.6)
Stylet length (St)	-	-	21.1 ± 0.4 (20.7-21.7)
Conus length	-	-	13.2 ± 0.5 912.8-13.9)
Stylet shaft + knob height	-	-	7.9 ± 0.0 (7.9-7.8)
Knob width	-	-	3.7 ± 0.9 (3.5-3.8)
Knob height	-	-	1.7 ± 0.1 (1.6-1.8)
DGO	-	-	5.4 ± 0.3 (5.0-7.8)
Median bulb valve length	-	-	5.8 ± 0.3 (5.4-6.1)
Median bulb width	-	-	9.9 ± 0.0 (9.9-9.9)
Isthmus length	-	-	15.4 ± 0.6 (15.0-16.3)
Pharynx length	95.0 ± 5.5 (85.1-102.0)	?	94.7 ± 6.1 (89-103.2)
Anterior end to excretory pore (Ep)	83.3 ± 7.3 (72.0-91.5)	?	73.7 ± 1.5 (72.2-75.2)
Max body width	13.9 ± 0.7 (12.8-14.7)	12.0	16.0 ± 0.6 (15.5-16.8)
Body width at anus	10.4 ± 0.5 (9.9-10.8)	8.5	10.5 ± 0.2 (10.3-10.7)
Anterior end to median bulb base	-	-	57.6 ± 3.5 (54.0-62.3)
Genital tract length	121.4 ± 12.4 (105.0-142.5)	73.0	-
Tail length	44.4 ± 5.4 (38.0-50.4)	43.0	30.6 ± 0.1 (30.5-30.6)
Stylet base (Stb) to median bulb valve base (v)	-	-	27.5 ± 2.2 (24.7-30.2)
Spicule length	20.4 ± 0.7 (19.5-21.2)	19.5	-
Gubernaculum length	4.0 ± 0.3 (3.5-4.5)	3.5	-
PERCENTAGES			
G or T	30.1 ± 3.3 (24.5-33.9)	20.5	-
St/L	-	-	1.3 ± 0.1 (1.2-1.4)
Ep/L	21.6 ± 1.1 (20.7-23.8)	?	24.2 ± 0.0 (24.1-24.2)
RATIOS			
a	29.1 ± 1.3 (26.6-30.3)	29.7	19.7 ± 1.7 (17.7-21.8)
b	4.2 ± 0.1 (4.0-4.3)	?	3.3 ± 0.1 (3.2-3.5)
c	9.1 ± 0.7 (8.5-10.6)	8.3	9.9 ± 0.2 (9.7-10.1)
c′	4.1 ± 0.3 (3.8-4.6)	5.1	2.9 ± 0.1 (2.8-2.9)

Table S4. Morphometrics of *Paratylenchus minutus* (paratypes) and paratypes of the closely related species *P. shenzhenensis* Wang, Xie, LI, Xu Yu & Wang, 2013 from China.

**Population**	Linford *et al*. (1949). Values in bold from Raski (1975)		Paratypes of *Paratylenchus shenzhenensis* Wang *et al.* (2013) China	
**Character**	♀ (fixed)	♂ (fixed)	♀ (fixed)	♂ (fixed)
n	17	3	41	22
L	267.0 (240.0-310.0)	258.0 (220.0-270.0)	275.0 ± 11.9 (249.0-302.0)	262 ± 12.2 (236.0-280.0)
Stylet length (St)	(16.0-21.0)	-	19.0 ± 0.7 (17.0-21.0)	-
Conus length	-	-	12.0 ± 0.6 (11.0-14.0)	-
Stylet shaft + knob height	**11.0 (10.0-12.0)**	-	6.0 ± 0.4 (5.0-7.0)	-
Knob width	-	-	2.0 ± 0.3 (2.0-3.0)	-
Knob height	-	-	1.5 ± 0.2 (1.0-2.0)	-
DGO	-	-	3.0 ± 0.6 (2.0-4.0)	-
Median bulb valve length	-	-	5.0 ± 0.7 (4.0-7.0)	-
Isthmus length	-	-	11.0 ± 1.3 (8.0-16.0)	-
Pharynx length	**64**	-	68.0 ± 0.3 (60.0-72.0)	-
Anterior end to excretory pore (Ep)	**56.0 (49.0-59.0)**	**54.0 (49.0-59.0)**	63.0 ± 2.3 (59.0-68.0)	58.0 ± 2.7 (53.0-63.0)
Max body width	**15**	-	13.0 ± 1.3 (10.0-15.0)	9.0 ± 0.5 (8.0-10.0)
Lateral field width	-	-	2.0 ± 0.1 (1.5-2.0)	-
Anterior end-vulva distance	**224**	-	226.0 ± 9.7 (207.0-250.0)	-
Vulva-tail terminus distance	**46**	-	-	-
Genital tract length	**127**	-	-	-
Vulva-anus distance	**29**	-	-	-
Tail length	**17**	(14.0-19.0)	18.0 ± 2.0 (15.0-24.0)	16.0 ± 1.1 (15.0-19.0)
Spicule length	-	(16.0-19.0)	-	17.0 ± 0.7 (16.0-19.0)
Gubernaculum length	-	**3.0 (2.0-4.0)**	-	3.0 ± 0.4 (2.0-4.0)
PERCENTAGES				
V	(80.0-84.0)	-	82.2 ± 0.8 (79.6-84.2)	-
G or T	-	**30.0 (24.0-33.0)**	-	29.1 ± 3.3 (20.7 -34.0)
St/L	-	-	6.8 ± 0.3 (6.1-7.9)	-
Ep/L	-	-	22.8 ± 0.8 (21.4-24.6)	22.1 ± 0.8 (20.5-23.4)
RATIOS				
a	19.1 (16.0-24.0)	24.4 (22.0-27.0)	22.2 ± 2.6 (17.4-28.1)	29.0 ± 2.1 (25.0-33.9)
b	3.7 (3.4-4.1)	3.8 (3.5-4.1)	4.0 ± 0.2 (3.7-4.4)	-
c	(12.0-18.0)	14.4 (12.0-19.0)	15.5 ± 1.5 (11.5-18.8)	16.1 ± 0.8 (14.8-17.7)
c′	-	-	2.5 ± 0.3 (2.0-3.2)	2.3 ± 0.2 (2.0 -2.7)

Table S5. Morphometrics of females of *Paratylenchus paralatescens* paratypes Munawar *et al.* (2018) from an undefined bamboo from China compared to those reported for *P. latescens* Raski (1976) from mesquite in Texas.

**Population**	Unspecified bamboo (as *P. paralatescens*) Munawar *et al.* (2018) China	*Prosopis* sp. (as *P. latescens*) Raski (1976) Texas
**Character**	♀ (fixed)	♀ (fixed)
n	26	19
L	290.4 ± 11.8 (271.1-308.1)	270.0 ± 16.9 (220.0-290.0)
Stylet length (St)	74.8 ± 2.4 (71.5-78.8)	75.0 (63.0-80.0)
Conus length	-	67.0 (58.0-74.0)
Stylet shaft + knob height	-	7.0 (5-10)
Median bulb valve length	-	9.0
Median bulb width	7.5 ± 0.6 (6.2-8.6)	-
Isthmus length	-	11.0
Pharynx length	125.6 ± 4.6 (118.7-133.5)	-
Anterior end to excretory pore (Ep)	68.8 ± 2.8 (62.7-73.4)	71.0 (64.0-82.0)
Max body width	11.5 ± 0.7 (10.3-12.7)	-
Body width at vulva	10.9 ± 0.6 (9.7-12.5)	-
Body width at anus	5.9 ± 0.5 (5.0-6.8)	-
Vulva-tail terminus distance	80.5 ± 3.4 (55.5-109.0)	-
Tail length	26.2 ± 2.5 (21.3-30.5)	-
PERCENTAGES		
V	70.1 ± 1.2 (68.2-72.9)	71.0 (68.0-73.0)
RATIOS		
a	25.3 ± 1.7 (22.4 -29.0)	19.0 (14.0-23.0)
b	2.3 ± 0.1 (2.2-2.5)	2.2 (1.7-2.5)
c	11.2 ± 1.1 (9.3-13.8)	13.0 (10-13)
c'	4.5 ± 0.4 (3.2-5.4)	-

Table S6. Morphometrics of males of *Paratylenchus paralatescens* populations from *Phyllostachys nigra* (Florida), *Phyllostachys bambusoides* (Florida) Troccoli *et al*. (2002) and *P. paralatescens* paratypes Munawar *et al.* (2018) from an undefined bamboo from China compared to those reported for *P. latescens* Raski (1976) from mesquite in Texas.

**Population**	*Phyllostachys nigra* (CD3396 – N20-01262-1) Florida		*Phyllostachys bambusoides* (as *P. latescens*) Troccoli *et al*. (2002) Florida	Unspecified bamboo (as *P. paralatescens*) Munawar *et al*. (2018) China	*Prosopis* sp. (as *P. latescens*) Raski (1976) Texas
**Character**	♂ (live)	♂ (fixed)	♂ (fixed)	♂ (fixed)	♂ (fixed)
n	4	2	7	4	8
L	305.1 ± 13.2 (287.0-318.7)	293.0, 322.0	298.0 ± 24.9 (278.0-348.0)	312.8 ±12.4 (300.3-324.3)	290.0 (230.0-250.0)
Pharynx length	79.3 ± 0.16 (71.0-86.1)	65.0, 82.1	-	90.4 ± 2.4 (87.8-93.4)	97.2
Anterior end to excretory pore (Ep)	66.0 ± 2.9 (63.3-70.2)	66.3, 69.8	65.0 ± 6.9 (56.0-76.0)	66.3 ± 1.9 (63.6-67.8)	65.0 (58.0-77.0)
Max body width	11.9 ± 0.7 (10.8-12.6)	10.3, 12.4	10.5 ± 0.5 (10.0-11.5)	10.4 ± 0.4 (10.1-10.9)	12.0
Body width at anus	9.1 ± 0.5 (8.5-9.9)	8.4, 8.4	7.5 ± 0.3 (7.5-8.0)	8.0 ± 0.7 (7.4-8.8)	-
Lateral field width	1.9 ± 0.0 (1.8-1.9)	1.9, 1.8	-	-	-
Genital tract length	112.4 ± 4.0 (108.0-116.8)	136.6, 136.6	118.0 ± 1.9 (111.0-124.0)	-	(40.0-49.0)
Tail length	25.8 ± 1.9 (23.2-27.7)	22.7, 27.0	26.5 ± 2.2 (24.0-30.5)	28.2 ± 1.5 (26.7-29.5)	26.9
Gubernaculum length	4.1 ± 0.2 (4.0-4.4)	4.2, 4.0	4.5. ± 0.2 (4.5-5.0)	4.5 ± 0.6 (4.0-5.3)	4.0
Spicule length	18.4 ± 0.4 (18.0-18.8)	18.0, 17.9	18.5 ± 0.7 (18.0-19.5)	18.1 ± 0.7 (17.5-18.9)	(40.0-49.0)
PERCENTAGES					
G or T	36.8 ± 1.7 (34.3-38.8)	47.3, 42.4	39.3 ± 2.8 (35.6-42.8)	31.5 ± 5.3 (23.8-35.7)	37.0 (27.0-47.0)
Ep/L	21.6 ± 1.0 (20.0-22.5)	22.6, 21.0	-	-	-
RATIOS					
a	25.7 ± 0.7 (24.8-26.5)	28.4, 25.9	28.6 ± 0.9 (27.6-30.3)	30.2 ± 2.0 (27.6 -32.1)	26.0 (20.0-29.0)
b	3.8 ± 0.1 (3.7-4.0)	4.5, 3.9	-	3.5 ± 5.3 (3.3-3.7)	3.5 (3.3-3.7)
c	11.8 ± 0.4 (11.4-12.3)	12.5, 11.1	11.5 ± 0.7 (10.5-12.6)	11.1 ± 0.8 (10.3-12.1)	12.0 (11.0-13.0)
c′	3.8 ± 0.1 (3.7-4.0)	2.7, 3.4	3.4 ± 0.3 (3.0-3.8)	3.6 ± 0.3 (3.3-4.0)	-

Table S7. Reference sequences of *Paratylenchus* species used in the present study.

**Species**	**Original species description reference**	**Type locality / host**	**Accession number of reference sequences**			**Comments**
			**D2-D3 of 28S rRNA gene**	**ITS rRNA gene**	***COI* gene**	
P. aciculus	Brown (1959)	Canada, Ontario, Blackburn / fowl bluegrass	MZ265071	MZ265008	MZ262212	Specimens collected from Jaén, Spain in *Pinus halepensis*, identified as *P. aciculus* were morphologically and molecularly characterized by Clavero-Camacho *et al.* (2021). **Molecular characterization of specimens collected from type host and locality is required.**
P. acti	Eroshenko (1978)	Russia, Sakhalin Island / pine	OQ749957	OQ749711	OQ755146	Specimens collected from Florida, USA in *Andropogon virginicus*, identified as *P. acti* were morphologically and molecularly characterized in this study. **Molecular characterization of specimens collected from type host and locality is required.**
P. aculentus	Brown (1959)	Canada, Saskatchewan, Lake Lenore / grass sod	MW413626	MW413588	MW421639	Specimens collected from Belgium in grasses, identified as *P. aculentus* were morphologically and molecularly characterized by Singh *et al.* (2021). **Molecular characterization of specimens collected from type host and locality is required.**
P. audriellus	Brown (1959)	Canada, Ontario, Orleans / white birch	KU291238	-	-	Specimens collected Kermanshah province, Iran, identified as *P. audriellus* were morphologically and molecularly characterized by Esmaeili *et al.* (2016). **Molecular characterization of specimens collected from type host and locality is required.**
P. amundseni	Bernard (1982)	USA, Alaska, Adak Island, Shagak Bay / *Elyrnus mollis*	MZ265076	MZ265012	MZ262215	Specimens collected from Jaén, Spain in *Pinus halepensis*, identified as *P. amundseni* were morphologically and molecularly characterized by Clavero-Camacho *et al.* (2021). **Molecular characterization of specimens collected from type host and locality is required.**
P. aquaticus	Merny (1966)	Ivory Coast, Béoumi / rice	KF242241 (type B) / OQ749952 (type C)	OQ749709 (type C)	MW411838 (type B) / OQ755157 (type C)	Specimens collected from Manhattan, Kansas identified as *P. aquaticus* type B and from Florida, USA identified as *P. aquaticus* type C were morphologically and molecularly characterized by Van den Berg *et al.* (2014) and by this study, repectively. **Molecular characterization of specimens collected from type host and locality is required.**
P. baldaccii	Raski (1975)	Italy, Sicily / grape	MW798290	MW798335	MW797012	Specimens collected from Sevilla, Spain in peach, identified as *P. baldaccii* were morphologically and molecularly characterized by Clavero-Camacho *et al.* (2021). **Molecular characterization of specimens collected from type host and locality is required.**
P. beltsvillensis	Kantor *et al.* (2021)	USA, Maryland, Beltsville / Virginia pine	MW413581	MW413587	-	Specimens collected from the type host and locality were morphologically and molecularly characterized by Kantor *et al.* (2021). **Molecular characterization of *COI* gene of specimens collected from type host and locality is required.**
P. bilineatus	Brzeski (1995)	Taiwan, Shuang Hai / bamboo	-	EU247525	-	Specimens collected from the type host in Taiwan were morphologically and molecularly characterized by Chen *et al.* (2008). **Molecular characterization of 28S rRNA and *COI* genes of specimens collected from type host and locality is required.**

P. borealis sp. n.	This study	USA, Alaska, Anchorage / unknown plants	OQ749978	OQ749710	MW421700	Specimens collected from the type host and locality were molecularly characterized in this study. **Molecular characterization of *COI* gene of specimens collected from type host and locality is required.**
P. canchicus	Mohilal & Dhanachand (2004)	India, Uttar Pradesh	ON873212	-	ON873964	Specimens collected from Málaga, Spain in maritimus pine, identified as *P. canchicus* were morphologically and molecularly characterized by Palomares-Rius *et al.* (2022b). **Molecular characterization of ITS rRNA gene of specimens collected from type host and locality is required.**
P. capitatus	Adams & Eichenmuller (1962)	USA, West Virginia / scarlet oak	-	-	MN710982	Specimens collected North Carolina, USA, identified as *P. capitatus* were molecularly characterized by Powers *et al.* (2021). **Molecular characterization of specimens collected from type host and locality is required.**
P. caravaquenus	Clavero-Camacho *et al.* (2021)	Spain, Murcia, Caravaca / pine	MW798270	MW798316	MW797003	Specimens collected from the type host and locality were morphologically and molecularly characterized by Clavero-Camacho *et al.* (2021).
P. chongqingensis	Li *et al*. (2019)	China, Chongqing / *Ophiopogon japonicus*	-	MK024368	-	Specimens collected from the type host and locality were morphologically and molecularly characterized by Li *et al.* (2019). **Molecular characterization of 28S rRNA and *COI* genes of specimens collected from type host and locality is required.**
P. coronatus	Colbran (1965)	Australia, Queensland / *Baloghia lucida*	MK506808	MK506795	-	Specimens collected from East Azerbayjan, Iran in *Crataegus* sp., identified as *P. coronatus* were morphologically and molecularly characterized by Mokaram Hesar *et al*. (2019). **Molecular characterization of specimens collected from type host and locality is required.**
P. dianthus	Jenkins & Taylor (1956)	USA, Maryland / *Dianthus caryophyllus*	KF242226	KF242271	MW411837	Specimens collected from Gauteng, South Africa in *Chrysanthemum*, identified as *P. dianthus* were morphologically and molecularly characterized by Van den Berg *et al.* (2014). **Molecular characterization of specimens collected from type host and locality is required.**
P. elachistus	Steiner (1949)	USA, Florida. Clewiston/ *Boehmeria nivea*	MW413629	MW413590	MW421643	Specimens collected from Belgium in grasses, identified as *P. elachistus* were morphologically and molecularly characterized by Singh *et al.* (2021). **Molecular characterization of specimens collected from type host and locality is required.**
P. enigmaticus	Munawar *et al*. (2021)	Canada, Alberta, Taber / potato field	MW282760	MW282772	MZ262222	Specimens collected from the type host and locality were morphologically and molecularly characterized by Munawar *et al*. (2021). **Molecular characterization of *COI* gene of specimens collected from type host and locality is required.**
P. goldeni	Raski (1975)	USA, North Carolina / *Buxus* sp.	OQ749969	OQ749700	OQ755152	Specimens collected from Florida, USA in *Ilex* sp, identified as *P. goldeni* were morphologically and molecularly characterized in this study. **Molecular characterization of specimens collected from type host and locality is required.**
P. goodeyi	Ostenbrink (1953)	The Netherlands, Arnhem / grasses	MW413631	MW413594	MW421647	Specimens collected from Belgium in grasses, identified as *P. goodeyi* were morphologically and molecularly characterized by Singh *et al.* (2021). **Molecular characterization of specimens collected from type host and locality is required.**
P. guangzhouensis	Wang *et al*. (2016)	China, Guangdong Province, Guangzhou / *Bambusa multiplex*	-	KT725625	-	Specimens collected from the type host and locality were morphologically and molecularly characterized by Wang et al. (2016). **Molecular characterization of 28S rRNA and *COI* genes of specimens collected from type host and locality is required.**
P. hamatus	Thorne & Allen (1950)	USA, California / fig tree	KF242212	KF242244	MW411821	Specimens collected from the type host and locality were morphologically and molecularly characterized by Van den Berg *et al.* (2014).
P. hamicaudatus	Cid del Prado Vera & Maggenti (1988)	USA, California, Marin County / Sequoia sempervirens	OQ749973	OQ749705	OQ755158	Specimens collected from the type host and locality were molecularly characterized in this study.

P. hawaiiensis sp. n.	This study	USA, Florida, Miami-Dade County / *Aechmea* sp.	OQ749977	KF242277	MW411845	Specimens collected from the type host and locality were molecularly characterized in this study. **Molecular characterization of ITS rRNA and *COI* genes of specimens collected from type host and locality is required.**
P. holdemani	Raski (1975)	El Salvador, Santa Anna / coffee	MW413639	MW413596	MW421653	Specimens collected from Belgium, grasses, identified as *P. holdemani* were morphologically and molecularly characterized by Singh *et al.* (2021). **Molecular characterization of specimens collected from type host and locality is required.**
P. idalimus	Raski (1962)	USA, California / *Ericameria palmeri*	KF242237	KF242275	MW411839	Specimens collected from Belgium, grasses, identified as *P. idalimus* were morphologically and molecularly characterized by Singh *et al.* (2021). Specimens collected from California, USA in grape were molecularly characterized by Van den Berg *et al.* (2014). **Molecular characterization of specimens collected from type host and locality is required.**
P. ilicis	Singh *et al.* (2022)	The Netherlands, Hilversum / *Ilex aquifolium*	MW413683	ON668073	MW421706	Specimens collected from the type host and locality were morphologically and molecularly characterized by Singh *et al.* (2022).
P. indalus	Clavero-Camacho *et al.* (2021)	Spain, Almería, Santa María de Nieva / almond	MW798273	MW798319	MW797005	Specimens collected from the type host and locality were morphologically and molecularly characterized by Clavero-Camacho *et al.* (2021).
P. israelensis	Raski (1973)	Israel, Shiller / plum	MW798301	MW798343	MW797019	Specimens collected from Cordoba, Spain in almond, identified as *P. israelensis* were morphologically and molecularly characterized by Clavero-Camacho *et al.* (2021). **Molecular characterization of specimens collected from type host and locality is required.**
P. jasmineae	Phani *et al*. (2019)	India, New Delhi / *Jasminum sambac*	MH588059	MH588060	-	Specimens collected from the type host and locality were morphologically and molecularly characterized by Phani *et al*. (2019). **Molecular characterization of *COI* gene of specimens collected from type host and locality is required.**
P. labiosus	Anderson & Kimpinski (1977)	Canada, Prince Edward Island, Souris / potato	MK506809	MK506796	-	Specimens collected from East Azerbayjan, Iran in *Malus* sp., identified as *P. labiosus* were morphologically and molecularly characterized by Mokaram Hesar *et al*. (2019). **Molecular characterization of specimens collected from type host and locality is required.**
P. lepidus	Raski (1975)	Sri Lanka / *Thea sinensis*	MK886692	MK886695	MT828831	Specimens collected from Zhejiang Province, China in *Elaeocarpus* sp. identified as *P. lepidus* were morphologically and molecularly characterized by Munawar *et al.* (2019). **Molecular characterization of specimens collected from type host and locality is required.**
P. leptos	Raski (1975)	Brazil, Nova Teutonia / *Piper* sp.	MW413645	-	MW421663	Specimens collected from Gera district, Ethiopia in coffee, identified as *P. leptos* were molecularly characterized by Singh *et al.* (2021). **Molecular characterization of specimens collected from type host and locality is required.**
P. macrodorus	Brzeski (1963)	Poland, Skierniewice / *Cichorium intybus*	MZ265108	MZ265034	MZ262239	Specimens collected from Córdoba, Spain in wild olive, identified as *P. macrodorus* were morphologically and molecularly characterized by Clavero-Camacho *et al.* (2021). **Molecular characterization of specimens collected from type host and locality is required.**
P. microdorus	Andrássy (1959)	Montenegro, Durmitor mountains / grasses	MW413654	MW413598	MW421666	Specimens collected from Belgium in grasses identified as *P. microdorus* were morphologically and molecularly characterized by Singh *et al.* (2021). **Molecular characterization of specimens collected from type host and locality is required.**
P. minor	Sharma *et al.* (1986)	India, Himachal Pradesh, Juneo / *Prunus persica*	MK660188	MK660189	-	Specimens collected from Zhejiang Province, China in *Pinus tabuliformis* identified as *P. minor* were morphologically and molecularly characterized by Munawar *et al.* (2019). **Molecular characterization of specimens collected from type host and locality is required.**

P. minutus	Linford *et al.* (1949)	USA, Hawaii, Oahu, Wahiawa / *Ananas comosus*	OQ749976	OQ749706	-	Specimens collected from Hawaii, USA in *Coffea* sp., identified as *P. minutus* were morphologically and molecularly characterized in this study. **Molecular characterization of *COI* gene of specimens collected from type host and locality is required.**
P. nainianus	Edward & Misra (1963)	India, Uttar Pradesh / *Psidium cattleianum*	ON873217	ON873186	ON873966	Specimens collected from Málaga, Spain in green heather, identified as *P. nainianus* were morphologically and molecularly characterized by Palomares-Rius *et al.* (2022b)
P. nanjingensis	Wang *et al.* (2016)	China, Jiangsu Province, Nanjing / *Pinus massoniana*	KR232932	KM366101	-	Specimens collected from the type host and locality were morphologically and molecularly characterized by Wang *et al.* (2016). **Molecular characterization of *COI* gene of specimens collected from type host and locality is required.**
P. nanus	Cobb (1923)	USA, Virginia / grasses	MW234450	MW234452	MW238474	Specimens collected from the type host and locality were morphologically and molecularly characterized by Subbotin *et al.* (2020).
P. nawadus	Khan *et al.* (1967)	India, Bihar / *Carissa* sp.	MN088373	-	-	Specimens collected from Behbahan, Khuzestan, Iran in *Gundelia tournefortii* identified as *P. nawadys* were morphologically and molecularly characterized by Mirbabaei *et al.* (2019). **Molecular characterization of specimens collected from type host and locality is required.**
P. neoamblycephalus	Geraert (1965)	Germany, Frauweiler / *Malus*	MW413660	MW413606	MW421675	Specimens collected from Belgium in cypress tree identified as *P. neoamblycephalus* were morphologically and molecularly characterized by Singh *et al.* (2021). **Molecular characterization of specimens collected from type host and locality is required.**
P. neonanus	Mathur *et al.* (1967)	India, Uttar Pradesh, Meerut / *Ziziphus* sp.	ON873226	-	ON873976	Specimens collected from Málaga, Spain in maritimus pine, identified as *P. neonanus* were morphologically and molecularly characterized by Palomares-Rius *et al.* (2022b) **Molecular characterization of specimens collected from type host and locality is required.**
P. neoprojectus	Wu & Hawn (1975)	Canada, Alberta / alfalfa	MW282762	MW282775	-	Specimens collected from Alberta, Canada in *Chenopodium* sp., identified as *P. neoprojectus* were morphologically and molecularly characterized by Munawar *et al.* (2021). **Molecular characterization of *COI* gene of specimens collected from type host and locality is required.**
P. pandatus	Raski (1976)	Nigeria, Katsina / grapefruit	MZ265116	MZ265041	MZ262247	Specimens collected from Caravaca, Murcia, Spain in *Pinus halepensis*, identified as *P. pandatus* were morphologically and molecularly characterized by Clavero-Camacho et al. (2021). **Molecular characterization of specimens collected from type host and locality is required.**
P. paraaonli	Palomares-Rius *et al.* (2022b)	Spain, Málaga, Casares/ maritimus pine	ON873200	ON873174	ON873946	Specimens collected from the type host and locality were morphologically and molecularly characterized by Palomares-Rius *et al.* (2022b)
P. paralatescens	Munawar *et al.* (2018)	China, Zhejiang Province, Anji County / bamboo trees	MH200616	MH200615	-	Specimens collected from the type host and locality were morphologically and molecularly characterized by Munawar *et al.* (2018). **Molecular characterization of *COI* gene of specimens collected from type host and locality is required.**
P. parastraeleni	Clavero-Camacho *et al.* (2021)	Spain, Jaén, Arroyo Frío / *Quercus faginea*	MZ265064	MZ265004	MZ262208	Specimens collected from the type host and locality were morphologically and molecularly characterized by Clavero-Camacho *et al.* (2021).
P. pedrami	Clavero-Camacho *et al.* (2021)	Spain, Córdoba / almond	MW798283	MW798329	MW797009	Specimens collected from the type host and locality were morphologically and molecularly characterized by Clavero-Camacho *et al.* (2021).
P. peraticus	Raski (1962)	Germany, Rehlinger Trausch / grapes	MK506805	MK506792	-	Specimens collected from Ardebil, Iran in *Prunus* sp., identified as *P. peraticus* were morphologically and molecularly characterized by Mokaram Hesar *et al.* (2019). **Molecular characterization of specimens collected from type host and locality is required.**
P. pestis	Thorne (1943)	USA, California, Santa Clara / *Juglans regia*	MK506802	MK506790	-	Specimens collected from East Azerbayjan, Iran in *Juglans regia*, identified as *P. pestis* were morphologically and molecularly characterized by Mokaram Hesar *et*

						al. (2019). **Molecular characterization of specimens collected from type host and locality is required.**
P. plesiostraeleni	Palomares-Rius *et al.* (2022b)	Spain, Málaga, Tolox,	ON873204	ON873179	ON873954	Specimens collected from the type host and locality were morphologically and molecularly characterized by Palomares-Rius *et al.* (2022b)
P. projectus	Jenkins (1956)	USA, Maryland, Upper Marlboro / grass	MT668712	MT668705	MW238473	Specimens collected from California, North Dakota, USA and South Africa identified as *P. projectus* were morphologically and molecularly characterized by Van den Berg *et al.* (2014) and Subbotin *et al.* (2020). Molecular characterization of specimens collected from type host and locality is required.
P. recisus	Siddiqi (1996)	Carimagua, Colombia / *Stylosanthes guianensis*	MZ265119	MZ265043	MZ262252	Specimens collected from Jaén, Spain in *Quercus faginea*, identified as *P. recisus* were morphologically and molecularly characterized by Clavero-Camacho *et al*. (2021). **Molecular characterization of specimens collected from type host and locality is required.**
P. roboris sp. n.	This study	USA, Florida / *Quercus virginiana*	OQ749956	OQ749714	OQ755148	Specimens collected from the type host and locality were morphologically and molecularly characterized in this study.
P. rostrocaudatus	Huang & Raski (1987)	Brazil, Fazenda Sossego / rice	KR270601	KR270604	-	Specimens collected from China in bamboo, identified as *P. rostrocaudatus* were morphologically and molecularly characterized by Zhuo *et al*. (2018). **Molecular characterization of specimens collected from type host and locality is required.**
P. salubris	Raski (1975b)	Brazil, Porto Seguro / *Theobroma cacao*	ON873233	-	ON873980	Specimens collected from Málaga, Spain in maritimus pine, identified as *P. salubris* were morphologically and molecularly characterized by Palomares-Rius *et al.* (2022b). **Molecular characterization of specimens collected from type host and locality is required.**
P. sheri	Raski (1973)	France, Digne / grasses	MZ265121	MZ265044	MZ262253	Specimens collected from Jaén, Spain in grasses, identified as *P. sheri* were morphologically and molecularly characterized by Clavero-Camacho et al. (2021). **Molecular characterization of specimens collected from type host and locality is required.**
P. similis	Khan *et al*. (1967)	India, Mysore, Dhadesugar / sorghum	MK506806	MK506793	-	Specimens collected from East Azerbayjan, Iran in *Prunus* sp., identified as *P. similis* were morphologically and molecularly characterized by Mokaram Hesar *et al*. (2019). **Molecular characterization of specimens collected from type host and locality is required.**
P. sinensis	Munawar *et al.* (2020)	China Zhejiang Province, Jiaxing City / white mulberry	MN129026	MN129021	-	Specimens collected from the type host and locality were morphologically and molecularly characterized by Munawar *et al.* (2020). **Molecular characterization of *COI* gene of specimens collected from type host and locality is required.**
P. straeleni	De Coninck (1931)	Belgium, Liège Province, High Fens / moss	MW413685	MW413624	MW421708	Specimens collected from California, USA and Belgium in grasses identified as *P. straeleni* were morphologically and molecularly characterized by Van den Berg *et al*. (2014) and Singh *et al*. (2021). **Molecular characterization of specimens collected from type host and locality is required.**
P. tateae	Wu & Townshend (1973)	Canada, Ontario / corn	MW282754	MW282766	MZ262262	Specimens collected from Southern Alberta, Canada in potato and Zaragoza, Spain in almond, identified as *P. tateae* were morphologically and molecularly characterized by Munawar *et al*., (2021) and Clavero-Camacho *et al.* (2021). **Molecular characterization of specimens collected from type host and locality is required.**
P. tenuicaudatus	Wu (1961)	Canada, Ontario, North Gowe / red clover	MW798306	MW798347	MW797021	Specimens collected from Murcia, Spain in almond, identified as *P. tenuicaudatus* were morphologically and molecularly characterized by Clavero-Camacho *et al.* (2021). **Molecular characterization of specimens collected from type host and locality is required.**
P. teres	Raski (1976)	Brazil, Espirito Santo / *Theobrama cacao*	MN088376	-	-	Specimens collected from Khuzestan, Iran in *Pistacia atlantica*, identified as *P. teres* were morphologically and molecularly characterized by Mirbabaei *et al.* (2019).

						**Molecular characterization of specimens collected from type host and locality is required.**
P. variabilis	Raski (1975)	USA, California / *Rhus trilobata*	MZ265127	MZ265051	MZ262265	Specimens collected from Cordoba, Spain in wild olive, identified as *P. variabilis* were morphologically and molecularly characterized by Clavero-Camacho *et al.* (2021). **Molecular characterization of specimens collected from type host and locality is required.**
P. veruculatus	Wu (1962)	Scotland, Kilmcolm / heather	MW798311	MW798350	MW797027	Specimens collected from Almería, Spain in almond, identified as *P. veruculatus* were morphologically and molecularly characterized by Clavero-Camacho *et al.* (2021). **Molecular characterization of specimens collected from type host and locality is required.**
P. verus	Brzeski (1995)	Mexico, Texcoco / forest	MZ265130	MZ265054	MZ262268	Specimens collected from Cordoba, Spain in wild olive, identified as *P. verus* were morphologically and molecularly characterized by Clavero-Camacho *et al.* (2021). **Molecular characterization of specimens collected from type host and locality is required.**
P. vitecus	Pramodini *et al*. (2006)	India, Manipur / *Vitex peduncularis*	MZ265136	MZ265059	MZ262272	Specimens collected from Cordoba, Spain in wild olive, identified as *P. verus* were morphologically and molecularly characterized by Clavero-Camacho *et al.* (2021). **Molecular characterization of specimens collected from type host and locality is required.**
P. zurgenerus	Clavero-Camacho *et al.* (2021)	Spain, Almería, Zurgena / almond	MW798286	MW798331	MW797010	Specimens collected from the type host and locality were morphologically and molecularly characterized by Clavero-Camacho *et al.* (2021).
P. wuae	Yu *et al*. (2016)	Canada, Ontario, Ottawa / cow parsnip	KM061782	KM061783	-	Specimens collected from the type host and locality were morphologically and molecularly characterized by Yu *et al*. (2016). **Molecular characterization of *COI* gene of specimens collected from type host and locality is required.**
